# Can floseal™ be applied safely during otologic surgery? Assessment of ototoxicity in a chinchilla animal model

**DOI:** 10.1186/s40463-017-0203-5

**Published:** 2017-03-29

**Authors:** Carol Nhan, Aren Bezdjian, Abdullah Alarfaj, Sam J. Daniel

**Affiliations:** 1 McGill Auditory Sciences Laboratory, Department of Otolaryngology, McGill University, McGill University Health Center, 1001 Boulevard Décarie, Montréal, QC H4A 3 J1 Canada; 2 McGill Auditory Sciences Laboratory, Department of Experimental Surgery, McGill University, McGill University Health Center, 1001 Boulevard Décarie, Montréal, QC H4A 3 J1 Canada; 3 McGill Auditory Sciences Laboratory, McGill University Health Center, 1001 Boulevard Décarie, Montréal, QC H4A 3 J1 Canada; 4 McGill Auditory Sciences Laboratory, The Montreal Children’s Hospital, Department of Otolaryngology, McGill University, McGill University Health Center, 1001 Boulevard Décarie, Montréal, QC H4A 3 J1 Canada

**Keywords:** Ototoxicity, Safety, Floseal™, Otic, Intratympanic, Chinchilla, Auditory brainstem response

## Abstract

**Background:**

In otologic surgery good visualization is paramount, and patients with bleeding diatheses or who need to be anti-coagulated can present a significant challenge. Here, we determine whether Floseal™, a hemostatic matrix, is ototoxic in a validated animal model.

**Methods:**

Nine chinchillas housed in the animal care facilities of the Montreal Children’s Hospital Research Institute were used for the study. After a myringotomy incision was made in each tympanic membrane, baseline auditory brainstem response measurements were performed at 8, 20, and 25 kHz. In each animal one ear was randomized to receive Floseal™ to the middle ear cavity, whereas the other ear served as the control and received 0.9% sodium chloride. Outcome measures included early (day 7) and late (day 30) auditory brainstem response, clinical evidence of facial nerve or vestibular disturbance and histological evidence of ototoxity.

**Results:**

There was no significant hearing threshold shift on auditory brainstem response across all tested frequencies for both experimental and control ear. No animals receiving Floseal™ developed facial or vestibular nerve dysfunction and there was no histological evidence of ototoxicity.

**Conclusion:**

Based on the preliminary ototoxicity assessment on nine chinchillas, transtympanic Floseal™ does not appear to be ototoxic. More studies are warranted to assess the safety and applicability of the product in humans.

## Background

Floseal™ is a hemostatic matrix consisting of bovine-derived gelatin matrix and human-derived thrombin isolated from pooled plasma [1]. The thrombin is reconstituted in sodium chloride solution and mixed with the gelatin matrix prior to application. The high concentrations of human thrombin convert fibrinogen into fibrin monomers to accelerate clot formation and the gelatin granules swell to produce a tamponade effect. While it has been used for nasal and sinus bleeding [2–4] its use in the middle ear has not been reported.

In otologic surgery good visualization is paramount, and patients with bleeding diatheses or who need to be anti-coagulated can present a significant challenge. In circumstances where traditional hemostatic agents such as epinephrine, oxymetazoline, or gelfoam are ineffective, Floseal™ may be an option in ensuring adequate hemostasis and to complete otologic surgery. The objective of the present study was to evaluate the ototoxicity of Floseal™ in a chinchilla animal model.

## Methods

### Animal care and ethics

The study received approval by the Animal Care Committee of the McGill University Health Centre Research Institute and was conducted at the McGill Auditory Sciences Laboratory in accordance with the guidelines of the Canadian Council for Animal Care. Reporting of the animal study was done following the ARRIVE guidelines. Nine female chinchillas (*C. Laniger*, Ryerson Chinchilla Ranch, OH) with normal baseline auditory brainstem-evoked response (ABR) thresholds were included in the study. The animals were housed in temperature and light (12 h day/night cycle) controlled rooms with free access to water and commercial food by the animal care research facilities of the Montreal Children’s Hospital Research Institute.

### Hearing evaluation

Hearing assessment was conducted by measuring auditory brainstem response (ABR) on Chinchilla anesthetized by 5% Isoflurane and maintained with 3% Isoflurane. Acoustic stimuli of 8,000, 20,000, and 25,000 Hz pure tone bursts were presented to the Chinchilla through insert earphones starting at 80 dB intensity and decreasing by 5 dB until a threshold was reached. A threshold was identified when three replicable waves were found. Hearing evaluations were performed at baseline, early (day 7) and late (day 30) after application of Floseal™.

### Transtympanic application of floseal™

A 10 mL syringe of Floseal™ Hemostatic Matrix (Baxter) was prepared according to product instructions for use immediately prior to injection.

Each of nine animals had one ear randomized as the experimental ear, while the contralateral ear served as a control. An incision in the anterio-inferior quadrant of the tympanic membrane was made and 0.3–0.7 mL of Floseal™ (until visualization through the tympanic membrane under the microscope showed that the middle ear was filled) was introduced into the experimental middle ear via a soft polyethylene tubing catheter. The same volume of 0.9% NaCl was instilled into the control ears. Examination under general anesthesia the following day confirmed the presence of product in the middle ear cavities.

### Post-euthanasia middle ear examination and scanning electron microscopy (SEM)

Thirty days after the application of Floseal™ all animals were euthanized. The middle ears of all animals were opened and examined grossly for bony or mucosal changes. The cochlea were dissected, separated into apex, middle and base, then fixed in 4% paraformaldehyde. Post-fixation staining with osmium tetroxide and graded dehydration with 30, 50, 70, 80, 90, and 100% alcohol was performed. Specimens were critical-point dried using Leica CPD 030, mounted, gold plated, and viewed using the Hitachi field emission electron microscopy (Hitachi S4700, Tokyo, Japan).

### Statistical analysis

Early (day 7) and late (day 30) shifts in ABR thresholds after application of Floseal™ were compared using paired T-test between the experimental and control ears across all three frequencies tested (8, 20, 25 kHz). A *P*-value ≤ 0.05 was considered statistically significant. Our sample size was calculated with a minimum absolute difference represented by the mean ABR threshold difference of 20 dB, a standard deviation of 16 dB, and an alpha of .10. The ears were grouped depending on the treatment received.

## Results

### Physical observations

All animals receiving Floseal™ intratympanically remained in good health until the end of the experiment. No sign of weight loss, facial paralysis, head tilt or disequilibrium were present.

### ABR Threshold shifts

There was no significant difference in ABR thresholds before application and at day 7 and day 30 after application for both control and experimental ears at all three frequencies tested. The highest threshold shift was following 30 days of injection in the experimental ear at 20 kHz (11.1 ± 3.4 dB, *p* = 0.65) and was not significantly different from the control ear (10.8 dB, *p* = 0.87). No significant hearing loss difference was observed at all frequencies and time points tested (Table 1).

**Table 1:** Mean Hearing Threshold (dB) Differences from Baseline and day 7 and 30 post Injection

	Day 7			Day 30		
	Frequency (in kHz)					
	8	20	25	8	20	25
Experimental (Floseal™)	−8.3 ± 5.3	8.9 ± 3.4	−7.5 ± 3.8	−2.8 ± 3.4	11.1 ± 3.4	−0.3 ± 3.4
Control (PBS)	−7.8 ± 5.1	7.5 ± 4.0	−1.9 ± 5.1	−1.1 ± 3.5	10.8 ± 4.3	−0.8 ± 4.9

*dB* decibels, *kHz* kilohertz, *PBS* phosphate buffered saline

Auditory brainstem response (ABR) threshold shifts in the control and experimental ears at 7 and 30 days post-application of Floseal™

### Gross assessment

After euthanasia and temporal bone dissection revealed two animals with thickened white effusions in the middle ear. One animal had both experimental and control ears affected while the other was only affected in the experimental ear. For the remaining animals no changes in the bulla were noted.

### Histology (Scanning electron microscopy)

**Fig. 1:**
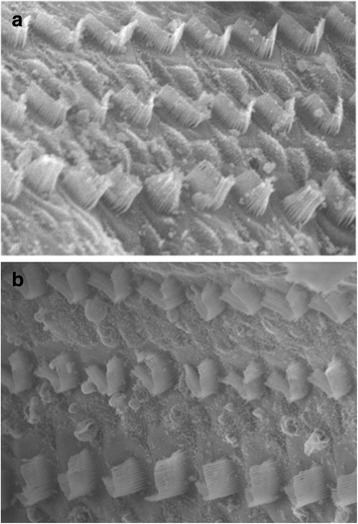
Scanning electron microscopy showing comparison between control (**a**) and experimental (**b**) histological micrographs

Three randomly selected pairs of cochlea were observed under SEM, which revealed no damage to the cochlear hair cells. The three rows of outer hair cells and the row of inner hair cells in the Organ of Corti were intact in both the control and experimental ears (Fig. 1).

## Discussion

Bleeding in middle ear surgery can significantly decrease visibility, potentially preventing successful completion of surgery. Most commonly, epinephrine or a decongestant such as oxymetazoline is used for homeostasis in middle ear surgery, however in cases where these agents are unsuccessful alternative options must be considered. Gelfoam is sometimes used as a packing agent in the middle ear, however its inability to conform to the spaces in the middle ear make it of limited use as an important hemostatic agent. Floseal™ is a commercial hemostatic agent composed of bovine-derived gelatin matrix and human-derived thrombin isolated from pooled plasma. It can be applied on wet, actively bleeding tissues, conforms to irregular surfaces and has been shown to be more effective than gelfoam in heparinized patients [5]. After application of the Floseal matrix, the gelatin particles swell and tamponade the bleeding as the gelatin matrix-thrombin composite slows the blood flow and provides exposure to a high concentration of thrombin, hastening clot formation [6]. Particularly in conditions where patients need to be anti-coagulated, such as having a metallic cardiac valve or stent it would be useful to know whether hemostatic agents such as Floseal™ can safely be used without causing ototoxicity. There have been reports questioning the possible ototoxicity of gelatin foam [7], however there is no clear scientific evidence of this; particularly when instilled in the middle ear.

Some agents such as gelatin foam have been suspected of contributing to conductive hearing loss due to scarring [8–10], however this has never been tested. Floseal™ appears as an acceptable option for hemostasis in the middle ear and surrounding structures. Our study is the first to demonstrate that Floseal™ applied to the middle ear is not ototoxic as evidenced by the ABR results on days 7 and 30 post-application as well as the electron microscopy of the cochlea. The non-significant threshold shifts found in Flosea™ and saline exposed ears likely resulted from an independent factor such as myringosclerosis from myringotomy and catheter manipulation during injection. Two animals were found to have thickened white effusions in the middle ear during temporal bone dissection. In one animal it was only in the experimental ear while in the other it was bilateral. Therefore, effusion occurred in 2 out of 9 ears (22%) receiving Floseal™ and 1 out of 9 control ears (11%). The underlying factor behind the found effusion is elusive; it could be associated with the surgical intervention or due to the presence of Floseal™ in the middle ear. Care should be taken in the use of Floseal™ in patients with atopy, particularly in those with a known allergy to gelatin products as this may lead to a life-threatening anaphylactic reaction [11].

The limitations of this study include the low sample size. The ototoxicity assessment of the present study was conducted in only nine animals. Also, although not evidenced by the physiological testing and histological analysis, the nature of effusion found in experimental ears should be further investigated.

## Conclusion

Based on the preliminary ototoxicity assessment on nine chinchillas, transtympanic Floseal™ does not appear to be ototoxic. More studies are warranted to assess the safety and applicability of the product in humans.
